# Pixel-Level Segmentation of Retinal Breaks in Ultra-Widefield Fundus Images with a PraNet-Based Machine Learning Model

**DOI:** 10.3390/s25185862

**Published:** 2025-09-19

**Authors:** Takuya Takayama, Tsubasa Uto, Taiki Tsuge, Yusuke Kondo, Hironobu Tampo, Mayumi Chiba, Toshikatsu Kaburaki, Yasuo Yanagi, Hidenori Takahashi

**Affiliations:** 1 Department of Ophthalmology, Jichi Medical University, Shimotsuke, Tochigi 329-0498, Japan; takayama.takuya@jichi.ac.jp (T.T.); ponta@jichi.ac.jp (H.T.); r-chiba@jichi.ac.jp (M.C.); kabutosi@jichi.ac.jp (T.K.); 2 DeepEyeVision Inc., Shimotsuke, Tochigi 329-0498, Japan; t-uto@deepeyevision.com (T.U.); t-tsuge@deepeyevision.com (T.T.); y-kondo@deepeyevision.com (Y.K.); 3 Department of Ophthalmology, Yokohama City University, Kanagawa 232-0023, Japan; yanagi.yas.wu@yokohama-cu.ac.jp; 4 Retina Research Group, Singapore Eye Research Institute, Singapore 168751, Singapore; 5 Center for Cyber Medicine Research, Tsukuba University, Ibaraki 305-8577, Japan

**Keywords:** retinal break, ultra-widefield fundus images, PraNet, segmentation, machine learning

## Abstract

Retinal breaks are critical lesions that can cause retinal detachment and vision loss if not detected and treated early. Automated, accurate delineation of retinal breaks in ultra-widefield fundus (UWF) images remains challenging. In this study, we developed and validated a deep learning segmentation model based on the PraNet architecture to localize retinal breaks in break-positive cases. We trained and evaluated the model using a dataset comprising 34,867 UWF images of 8083 cases. Performance was assessed using image-level segmentation metrics, including accuracy, precision, recall, Intersection over Union (IoU), dice score, and centroid distance score. The model achieved an accuracy of 0.996, precision of 0.635, recall of 0.756, IoU of 0.539, dice score of 0.652, and centroid distance score of 0.081. To our knowledge, this is the first study to present pixel-level segmentation of retinal breaks in UWF images using deep learning. The proposed PraNet-based model showed high accuracy and robust segmentation performance, highlighting its potential for clinical application.

## 1. Introduction

Rhegmatogenous retinal detachment (RRD) is a severe, vision-threatening disorder that arises from retinal breaks [1]. These defects allow liquefied vitreous humor to pass into the subretinal space, resulting in the separation of the neurosensory retina from the underlying retinal pigment epithelium (RPE) and subsequent retinal detachment. Although the prevalence of retinal breaks is estimated to be approximately 6% [2], many patients remain asymptomatic until RRD occurs [3]. Notably, more than 50% of untreated retinal breaks eventually progress to RRD due to sustained vitreoretinal traction [4].

Laser photocoagulation is the standard treatment for retinal breaks; however, the development of new retinal breaks following laser therapy has been reported in 12.5% to 13.7% of cases, and 14.5% of patients required additional laser procedures to treat the original break [5,6,7]. Furthermore, progression to retinal detachment has been observed in 5.5–6.9% of case [5,6,7]. If the condition progresses to RRD, surgical intervention such as pars plana vitrectomy or scleral buckling is necessary to reattach the retina and restore anatomical integrity. Given these risks, accurate delineation—not merely detection—of retinal breaks is essential for optimizing treatment strategies and preventing retinal detachment.

Recent advancements in ultra-widefield-fundus (UWF) imaging have significantly improved the visualization of the peripheral retina. UWF imaging can capture up to 200 degrees of the retina in a single non-mydriatic fundus image, encompassing approximately 80% of the total retinal area [8]. This technology enables more comprehensive assessment and holds promise for the delineation of peripheral retinal lesions, including retinal breaks.

Artificial intelligence (AI), particularly deep learning, has achieved high accuracy in classification tasks for various retinal diseases such as diabetic retinopathy [9], age-related macular degeneration [10], and glaucoma [11]. Recent studies have further demonstrated the potential of deep learning for prediction, segmentation, and classification of retinal diseases, including approaches such as hyperspectral image analysis for diabetic retinopathy and novel vision transformer integration for glaucoma detection [12,13,14]. In contrast, most prior studies [15,16,17,18,19,20] on retinal breaks have focused on image-level classification, reporting metrics such as sensitivity, specificity, and area under the receiver-operating characteristic curve (AUC). While these methods are effective for screening, they provide limited spatial information, and their ability to precisely delineate the boundaries of lesions is inadequate. Consequently, there exists an unmet clinical need for AI-based retinal break analysis that enables accurate lesion contouring.

In contrast, segmentation task directly outputs lesion contours, enabling detailed morphological assessment and supporting precise therapeutic targeting [21,22]. Segmentation performance is better reflected by spatial overlap metrics such as the Dice coefficient and intersection-over-union (IoU).

Among these architectures, PraNet has emerged as a high-performing model originally developed for polyp segmentation in gastrointestinal endoscopy [23]. PraNet incorporates a reverse attention module, allowing it to focus on subtle and irregular lesion patterns, making it well suited for medical image segmentation tasks. Our laboratory has previously developed a PraNet-based deep learning model for estimating non-perfusion areas in UWF images [24]. This approach demonstrated that PraNet architecture, with its reverse attention module, is effective for segmenting retinal abnormalities in UWF images.

The aim of this study is to develop and validate a PraNet-based deep learning model for pixel-level segmentation of retinal breaks in UWF images, directly addressing the unmet clinical need for precise lesion delineation. Unlike prior studies, our approach leverages a large, unfiltered, real-world dataset, retaining challenging cases with image artifacts, variable quality, and diverse lesion morphology, to ensure clinically robust contour delineation.

The main contributions of this work are as follows:

Novel application of PraNet architecture for fine-grained segmentation of retinal breaks—to our knowledge, the first report to demonstrate pixel-level delineation of this lesion type.

- Emphasis on the clinical significance of pixel-level delineation—enabling precise treatment planning and prophylactic interventions, and addressing the unmet clinical need for AI-based retinal break analysis.
- Training on a large, diverse, unfiltered dataset (34,867 UWF images) without excluding poor-quality or artifact-laden cases, thereby enhancing real-world applicability.
- Robust segmentation performance, achieving high Dice coefficient and IoU across heterogeneous cases, validated on an independent clinical test set.

## 2. Materials and Methods

### 2.1. Dataset

We retrospectively collected all UWF images obtained at Jichi Medical University Hospital, Saito Eye Clinic, Takahashi Eye Clinic, and Totsuka Eye Clinic between 2018 and 2021. All images were acquired using the Optos California retinal imaging system (Optos California, Nikon, Tokyo, Japan). No exclusion criteria were applied, allowing for a comprehensive dataset that reflects real-world clinical image variability—including peripheral artifacts, variable image quality, and complex cases. The entire dataset was randomly divided into three mutually exclusive subsets with no participant overlap. To rigorously evaluate segmentation and delineation performance, only images containing retinal breaks were included in the validation and test sets, ensuring that boundary contouring accuracy—not mere presence/absence—was measured in clinically relevant cases. Images without retinal breaks were used exclusively for training, addressing class imbalance and promoting model generalization. The dataset used in this study was accessed for research purposes between 1 June 2024, and 27 December 2024. During data collection and analysis, the authors had no access to any personally identifiable information. All data were fully anonymized before use.

### 2.2. Annotation

Retinal break regions were manually annotated at the pixel level by an experienced ophthalmic photographer (H Tam) using MENOU-TE version 1.36.300.0 (MENOU, Inc., Tokyo, Japan), an annotation software designed for medical imaging. An experienced vitreous surgeon (H Tak) checked the annotations. All annotated images were taken at Jichi Medical University only.

### 2.3. Data Preprocessing and Augmentation

In this study, we utilized automatically reconstructed pseudo-color UWF images for data preprocessing and model development. The original images had a resolution of 4000 × 4000 pixels. First, the margins were removed by cropping the central 3072 × 3072 pixels. The cropped images were then resized to 1536 × 1536 pixels for model input.

Data augmentation was applied to the input images as follows: random translations within ±10% horizontally and vertically, followed by random rotations within ±180 degrees. Next, random horizontal and vertical flips were performed. Additionally, the aspect ratio was varied between 0.9 and 1.1 while randomly cropping and rescaling portions of the image. Finally, brightness, contrast, and saturation were randomly adjusted to enhance visual diversity.

### 2.4. Deep Learning Model Development

**Figure 1:**
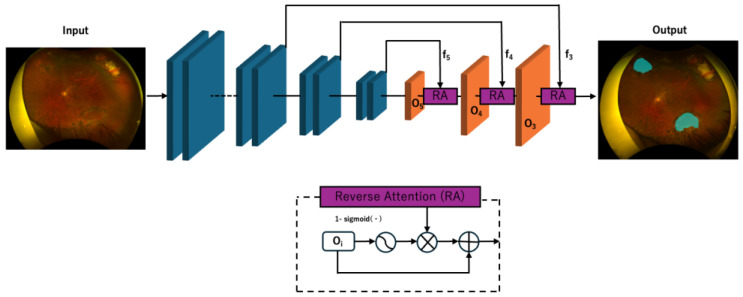
PraNet Diagram.

We adopted the PraNet architecture for delineating retinal breaks. The PraNet architecture consists of a backbone feature extractor (Res2Net-based), followed by a Parallel Partial Decoder (PPD) that aggregates high-level semantic features in parallel to generate a coarse global lesion map. Subsequently, a series of Reverse Attention (RA) modules are applied, each leveraging the prior map to recursively refine the segmentation boundaries by learning to distinguish subtle lesion edges and correcting misaligned predictions. Deep supervision is adopted for each intermediate output in addition to the global map, leading to more robust and boundary-aware segmentation. Weighted IoU and binary cross-entropy losses are used for training, placing greater emphasis on challenging pixels and edge regions. The overall workflow of the PraNet architecture used in this study is illustrated in Figure 1.

Input images were resized to 1536 × 1536 pixels, and all RGB channels were utilized. Deep learning model training and validation were conducted using PyTorch 1.13.1+cu116 and Torchvision 0.14.1+cu116, with CUDA 11.6 support. All training and validation were performed on a computing environment equipped with eight NVIDIA A100 GPUs, each with 80 GB of memory.

### 2.5. Hyperparameter Optimization

To enhance model performance, we performed a systematic hyperparameter optimization using Optuna (v3.0), an open-source framework for automated hyperparameter tuning. The optimization objective was to maximize the Dice coefficient on the validation dataset. For each trial, the model was trained for a fixed number of epochs. When the Dice coefficient plateaued, the trial was terminated and a new set of hyperparameters was evaluated.

**Figure 2:**
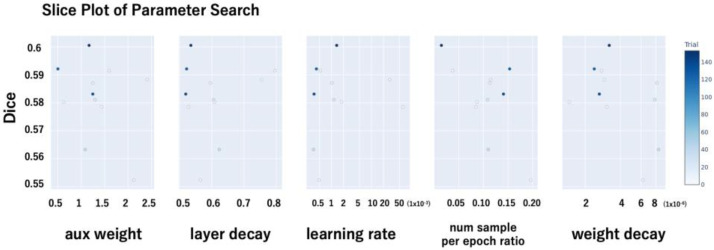
Slice plots of hyperparameter optimization trials. The vertical axis represents the Dice coefficient for each trial. Each subplot visualizes the relationship between a specific hyperparameter and the corresponding Dice coefficient (objective value) during hyperparameter optimization with Optuna. Each dot represents one trial, with color intensity indicating the trial number (darker colors denote later trials). From left to right, the subplots show: auxiliary loss weight (aux_weight), layer-wise learning rate decay (layer_decay), initial learning rate (lr), ratio of negative to positive samples per epoch (num_sample_per_epoch_ratio), and weight decay (weight_decay). These plots reveal the influence of each hyperparameter on model performance and guide the selection of optimal values based on Dice coefficient maximization.

The following hyperparameters were included in the search space:

- Auxiliary loss weight (aux weight): This parameter controls the contribution of the auxiliary loss in the PraNet architecture, which is intended to facilitate learning by providing additional supervision to intermediate layers.
- Layer-wise learning rate decay (layer_decay): A decay factor was applied to assign smaller learning rates to deeper layers, thereby preserving the pretrained weights of the backbone network.
- Initial learning rate (lr): The base learning rate used during training.
- Negative sample ratio per epoch (num_sample_per_epoch_ratio): To address class imbalance—specifically, the lower prevalence of retinal break images—this parameter defined the number of negative (non-break) images randomly sampled per epoch relative to the number of positive (break) images. For instance, a ratio of 0.5 implies that for every 500 positive images, 250 negative images were sampled per epoch.
- Weight decay: The L2 regularization coefficient used to mitigate overfitting.
- The hyperparameter search was conducted in a distributed manner. The optimal hyperparameter configuration was selected based on the trial that achieved the highest Dice coefficient (Figure 2).

### 2.6. Performance Evaluation

#### 2.6.1. Primary Outcome—Pixel-Level Segmentation Performance

The primary outcome was pixel-level segmentation performance, reflecting the precision of retinal break boundary delineation essential for clinical tasks such as laser photocoagulation planning, lesion monitoring, and reproducible documentation. Evaluation metrics included the following:

- Accuracy: the proportion of all pixels (lesion and background) correctly classified by the model. This metric was calculated as (TP + TN)/(TP + TN + FP + FN), where TP is true positive pixels, TN is true negative pixels, FP is false positive pixels, and FN is false negative pixels.
- Precision: the proportion of pixels predicted as lesion that were truly lesion pixels, calculated as TP/(TP + FP). This reflects the model’s ability to avoid false positives in the lesion class.
- Recall: the proportion of true lesion pixels that were correctly identified, calculated as TP/(TP + FN). This reflects the model’s ability to detect lesion pixels without omission.
- IoU: the ratio of the area of overlap to the area of union between the predicted lesion mask and the ground truth mask, calculated as TP/(TP + FP + FN). IoU measures spatial overlap and is less affected by pixel counts than overall accuracy.
- Dice score: the harmonic mean of precision and recall for the lesion class, calculated as 2 × TP/(2 × TP + FP + FN). The Dice score emphasizes correct prediction of lesion pixels and is widely used as a primary segmentation quality metric.
- Centroid distance score: the Euclidean distance (in pixels) between the centroid of the predicted lesion mask and the centroid of the corresponding ground truth mask. The centroid of each mask was computed as the mean (x, y) coordinate of all pixels within that mask. The distance was then normalized by the image width (1536 pixels) to yield a unitless value between 0 and 1. Lower scores indicate greater spatial localization accuracy, independent of lesion size.

#### 2.6.2. Secondary Outcome—Lesion-Wise Detection Performance

As a secondary analysis, we performed lesion-wise detection evaluation. A predicted region was counted as a true positive if it overlapped with an annotated lesion, and a strict one-to-one matching rule was applied for each case. In lesion-wise evaluation, a predicted region was considered a true positive if IoU > 0 with the annotated region. This inclusive criterion was adopted to capture all instances where the model successfully localized any part of the break, regardless of the precision of boundary overlap, given the small and irregular morphology of many lesions in the dataset. Sensitivity and positive predictive value (PPV) were then calculated. Because the validation and test sets contained only break-positive images, image-level specificity, false-positive rate, and ROC/AUC analyses were not performed. These metrics are reserved for future work incorporating mixed positive/negative evaluation sets.

All statistical analyses were conducted using Python (version 3.10; Python Software Foundation, Beaverton, OR, USA).

## 3. Results

### 3.1. Dataset Composition

The dataset comprised 8083 cases, 15,188 eyes, and 34,867 images. Of these, 960 images exhibited retinal breaks, while 33,907 images did not. Basically, images with retinal breaks were analyzed using 10-folds cross validation. 806 images for training, 81 for validation, and 73 for testing. Images without retinal breaks were partially used in training, the ratio of using the negative images was adjusted by Optuna and the final ratio was 1.5%. To align with the primary outcome of this study—precise pixel-level boundary segmentation of retinal breaks—the validation and test sets were intentionally composed exclusively of positive cases. This design ensured that evaluation metrics directly reflected the model’s ability to delineate lesion contours rather than being inflated by the presence of easy-to-classify negative images.

Negative (no-break) images were included only in the training set to address class imbalance during model development and to enhance generalization. This resulted in an inherently imbalanced dataset across the training/validation/test phases, which was a deliberate choice to rigorously assess segmentation accuracy under clinically relevant conditions. The dataset split was performed randomly with no participant overlap between subsets. Details are provided in Table 1.

### 3.2. Pixel-Level Segmentation Performance

The PraNet-based AI model’s pixel-level segmentation performance on the test set is summarized in Table 2. Mean accuracy was 0.996, with a precision of 0.635 and recall of 0.756. Spatial overlap measures included an IoU of 0.539 and a Dice score of 0.652, indicating moderate contour agreement. The mean centroid distance score was 0.081, reflecting good lesion localization precision.

### 3.3. Representative Segmentation Examples

**Figure 3:**
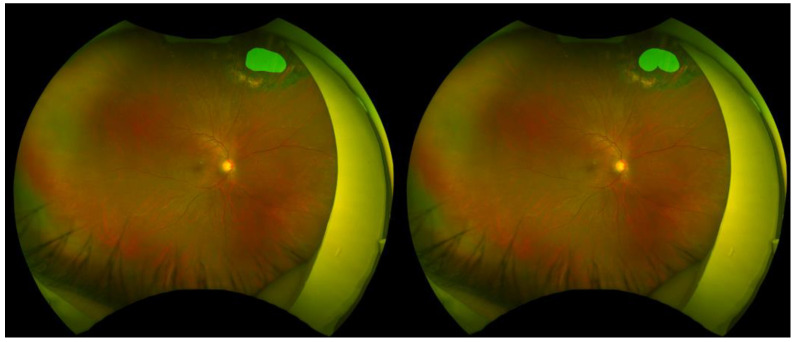
High-performing case.

**Figure 4:**
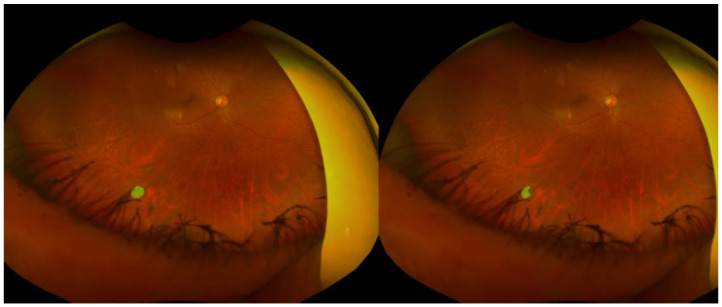
Moderately performing case.

**Figure 5:**
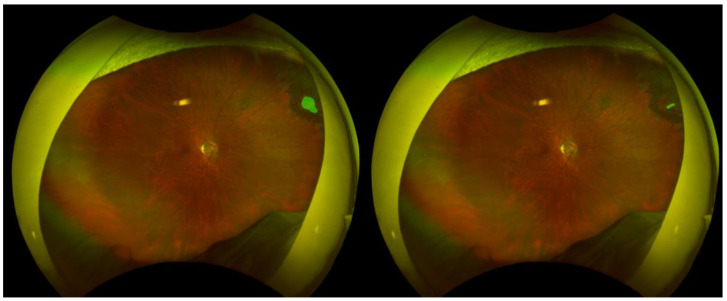
Poorly performing case.

Representative examples of retinal break segmentation are shown in Figure 3, Figure 4 and Figure 5, illustrating cases with high, moderate, and low segmentation performance as determined by pixel-wise and spatial accuracy metrics. Each figure includes the ground truth annotation (left) and the corresponding AI-generated segmentation mask (right).

This case achieved excellent segmentation performance, with an accuracy of 0.999, a precision of 0.920, a recall of 0.969, an IoU of 0.894, a dice score of 0.944, and a centroid distance score of 0.0014. Despite the presence of laser photocoagulation scars surrounding the retinal break, the model achieved highly accurate delineation.

This case achieved an accuracy of 0.999, a precision of 0.622, a recall of 0.985, an IoU of 0.616, a dice score of 0.763, and a centroid distance score of 0.0033. Eyelash artifacts partially overlapped the lesion area. Although the recall was high, the lower precision and IoU indicated that the model failed to accurately delineate part of the retinal break.

Although this case showed a high accuracy of 0.999, segmentation performance was limited, with a precision of 0.134, a recall of 0.976, an IoU of 0.134, a dice score of 0.236, and a centroid distance score of 0.0064. Similar to Figure 2, the retinal break was surrounded by laser photocoagulation scars, but only a small portion of the lesion was delineated by the model.

### 3.4. Background Data—Distribution of Breaks in Test Set

The test set contained 73 ultra-widefield fundus images with a total of 104 manually annotated retinal breaks (average: 1.425 ± 0.809 per image). The AI model predicted 131 retinal breaks (average: 1.699 ± 1.310 per image), as shown in Table 3.

### 3.5. Lesion-Wise Detection Performance

As a secondary analysis for comparability with prior detection-based AI studies, lesion-wise evaluation was conducted. A predicted region was counted as a true positive if it overlapped with an annotated lesion; a strict one-to-one matching was applied. Of the AI-predicted lesions, 92 were true positives, 32 were false positives, and 12 were false negatives. True negatives were not applicable in this analysis because the test set was intentionally composed of only break-positive cases, consistent with the study’s primary outcome of pixel-level segmentation performance evaluation. Under these conditions, sensitivity was 0.885 and PPV was 0.742 (Table 4). These values reflect lesion-level detection ability but do not capture the fine-grained contour accuracy required for clinical treatment planning.

## 4. Discussion

In this study, we present a deep learning model based on PraNet for the detection and pixel-level localization of retinal breaks in UWF images. Previous studies using artificial intelligence for the classification of retinal detachment and breaks have primarily focused on image-level performance metrics, such as the AUC [15,16,19,25]. These studies employed architectures such as EfficientNet and achieved high discriminative performance (AUCs ranging from 0.913 to 0.975); however, they lacked spatial interpretability. In particular, post hoc class activation maps (CAMs) often failed to accurately delineate retinal lesions [16].

Several studies have demonstrated the high diagnostic accuracy of AI systems in identifying retinal breaks. For instance, a model trained to classify UWF images containing lattice degeneration and/or retinal breaks achieved an AUC of 0.999, with sensitivity and specificity exceeding 98%. Another approach utilizing a YOLO v3 architecture successfully localized retinal breaks, yielding an AUC of 0.957 and a per-object average precision of 0.840. These systems have also outperformed general ophthalmologists in both sensitivity and agreement with expert labels, and interpretability has been enhanced through techniques such as Grad-CAM heatmaps [25,26]. Additionally, Li et al. attempted anatomical region-based localization by dividing UWF images into 48 segments and applying attention modulation modules (AMMs) [17]. However, retinal pathologies frequently present with small spatial footprints, low contrast, and heterogeneous morphologies, characteristics that make them particularly prone to being missed or misclassified by region-based detection methods. Their approach lacked the spatial resolution necessary to delineate lesion contours with clinical precision.

In contrast, our model enables precise pixel-level segmentation of retinal breaks, achieving high segmentation accuracy (mean Dice 0.652, mean IoU 0.539) and robust image-level localization (accuracy 0.996), underscoring the feasibility of automated, fine-grained boundary extraction. To our knowledge, this is the first report to demonstrate pixel-level segmentation of retinal breaks using deep learning. Such pixel-level localization is crucial for accurate diagnosis, individualized treatment planning, and effective monitoring as it enables detailed characterization of lesion morphology.

PraNet’s distinguishing feature lies in its reverse attention mechanism, which, unlike traditional attention models that emphasize lesion regions, learns to suppress background areas, thereby enhancing edge-level features [23,27]. This is particularly effective in medical images with subtle lesion boundaries. Additionally, PraNet incorporates a Parallel Partial Decoder (PPD) that integrates high-level features in parallel, enabling adaptation to lesions with varied morphology. Our previous work has shown the utility of AI-based approaches for detecting nonperfusion areas in UWF images [24], and this study further demonstrates PraNet’s applicability for detecting retinal breaks at a fine-grained level. Compared to region-based models such as those utilizing AMM, PraNet appears better suited for lesions with ambiguous boundaries and heterogeneous appearance.

Another important distinction of our study is its dataset inclusivity. Previous models often excluded cases with poor image quality, shallow detachments, or peripheral artifacts, and manually cropped “irrelevant” regions such as image corners [15,16,19,20,25,26]. Such pre-filtering may inflate performance under ideal conditions but limit real-world applicability. A prior study analysed misclassified images and identified that the common sources of error were lesions such as lesions partially obscured by eyelashes or regions with features resembling lattice degeneration. These issues could potentially be mitigated by augmenting the training dataset with a greater number of such challenging cases [26]. Our model was trained on unfiltered UWF images without exclusion criteria, thereby reflecting real-world clinical variability and offering a more robust evaluation of performance.

This study has several limitations related to study design and dataset composition. First, ground truth annotations were created by a single experienced ophthalmic photographer, which may introduce inter-observer variability. Consensus labeling by multiple experts could further enhance annotation quality. Second, the proportion of negative images included in the training dataset converged to nearly zero during optimization with Optuna, likely because validation was performed exclusively using fundus images from eyes with retinal breaks. While this approach is appropriate for the present study, which aimed to achieve accurate pixel-by-pixel detection of retinal breaks in eyes already known to have breaks, caution should be exercised regarding potential false positives when applying this algorithm to the general population. Third, the validation and test sets contained only break-positive cases, by design, to isolate and evaluate pixel-level segmentation accuracy in clinically relevant positives. As such, image-level specificity, false-positive rate, and ROC/AUC analysis were beyond the scope of this primary analysis and will be addressed in future studies incorporating mixed positive/negative evaluation sets. Finally, UWF images cover approximately 80% of the retina [8], which means retinal breaks located outside the imaging field or at the peripheral edges may be missed. Therefore, the AI model developed in this study cannot fully replace ophthalmologists’ examinations and should be positioned as an assistive tool to improve diagnostic accuracy and efficiency.

In addition, there are limitations related to model architecture, evaluation methodology, and image quality. While the inclusion of all image types improves external validity, it also introduces noise due to poor-quality or artifact-laden images, potentially affecting performance. The present study did not include direct performance comparisons with other established segmentation architectures (e.g., U-Net, DeepLabv3+). Therefore, while PraNet demonstrated robust performance, its relative advantage over alternative architectures remains to be validated in future work. For lesion-wise evaluation, a predicted region was defined as a true positive if the IoU with the ground-truth mask was greater than 0. This inclusive threshold was intentionally adopted to capture all cases where the model correctly localized any part of a lesion, given the small size, irregular shape, and low contrast of many retinal breaks in the dataset. However, this approach may overestimate detection performance compared with using higher IoU thresholds (e.g., 0.3 or 0.5), and future work should explore performance across multiple thresholds. Lastly, although segmentation performance was quantitatively assessed using IoU and Dice coefficients, the clinical utility of the model—such as improvements in diagnostic workflow or decision-making—was not evaluated. Future studies should include prospective trials to assess clinical impact.

## 5. Conclusions

In conclusion, we developed and validated a deep learning model based on the PraNet architecture for the pixel-level detection and localization of retinal breaks in UWF images. The PraNet model achieved high accuracy and demonstrated robust segmentation performance, even in challenging cases with ambiguous lesion boundaries. Our findings suggest that this method is useful for the precise delineation of retinal breaks and holds promise as a clinically applicable tool to improve patient outcomes, particularly in cases undergoing treatment for retinal breaks. Future work should focus on external validation across multiple institutions and the refinement of annotation strategies to further improve the model’s performance and generalizability.

## Figures and Tables

**Table 1:** Composition of the Ultra-Widefield Image Dataset.

Dataset	Retinal Break	No Retinal Break
Training	806	33,907 (finally 1.5% were used)
Validation	81	0
Test	73	0
Total	960	33,907

**Table 2:** Pixel-level image-wise segmentation performance metrics (test set).

Metric	Mean ± SD	Median
Accuracy	0.996 ± 0.010	0.999
Precision	0.635 ± 0.301	0.760
Recall	0.756 ± 0.281	0.864
IoU	0.539 ± 0.277	0.616
Dice score	0.652 ± 0.282	0.763
Centroid distance score	0.081 ± 0.170	0.013

**Table 3:** Distribution of Retinal Breaks in Test Dataset (per 73 Fundus Images).

Category	Total Number of Retinal Breaks	Average per Image (±SD)
AI-Predicted Retinal Breaks	131	1.699 ± 1.310
Annotated Retinal Breaks	104	1.425 ± 0.809

**Table 4:** Lesion-wise Detection Performance and Confusion Matrix.

	Predicted Positive	Predicted Negative
Actual Positive	92	12
Actual Negative	32	—
Metric	Value	
Sensitivity (Recall)	0.885	
Positive Predictive Value (PPV)	0.742	

## Data Availability

The test UWF image datasets used in this study are publicly available on Figshare (https://doi.org/10.6084/m9.figshare.29470040, accessed on 8 June 2025). The full dataset is pseudonymized and may be shared upon approval by the Ethics Committee of Jichi Medical University following a reasonable request for collaborative research. Here, “pseudonymized” refers to data processed such that individuals cannot be identified without the use of separately kept correspondence tables.

## Abbreviations

The following abbreviations are used in this manuscript:

UWF	Ultra-Widefield Fundus
IoU	Intersection over Union
PPV	Positive Predictive Value
RRD	Rhegmatogenous Retinal Detachment
RPE	Retinal Pigment Epithelium
AI	Artificial Intelligence
DLS	Deep Learning Systems
CNN	Convolutional Neural Network
AUC	Area Under the Curve
CAMs	Class Activation Maps
AMMs	Attention Modulation Modules
PPD	Parallel Partial Decoder
